# Mediterranean Diet Adherence and Screening Indicators of Gambling-Related Problems in a Sample of Young Adults: An Exploratory Cross-Sectional Analysis of the EVA-Adic Study

**DOI:** 10.3390/nu18172919

**Published:** 2026-09-06

**Authors:** Cristina Saldaña-Ruiz, Leticia Gómez-Sánchez, Alberto Vicente-Prieto, Sara Vicente-Gabriel, Cristina Lugones-Sánchez, Susana González-Sánchez, Sandra Conde-Martín, Marta Gómez-Sánchez, Manuel A. Gómez-Marcos

**Affiliations:** 1 Unidad de Investigación en Atención Primaria de Salamanca (APISAL), Gerencia de Atención Primaria de Salamanca, Gerencia Regional de Salud de Castilla y León (SACyL), Avenida de Portugal 83, 37005 Salamanca, Spain; csaldanar@saludcastillayleon.es (C.S.-R.); albertovicente94@gmail.com (A.V.-P.); u127891@usal.es (S.V.-G.); cristinals@usal.es (C.L.-S.);; 2 Instituto de Investigación Biomédica de Salamanca (IBSAL), Paseo de San Vicente, 58-182, 37007 Salamanca, Spain; 3 Departamento de Medicina, Facultad de Medicina, Universidad de Salamanca, Calle Alfonso X el Sabio s/n, 37007 Salamanca, Spain; 4 Servicio de Emergencias, Hospital Universitario de La Paz, Paseo de la Castellana, 261, 28046 Madrid, Spain; leticiagmzsnchz@gmail.com; 5 Red de Investigación en Cronicidad, Atención Primaria y Promoción de la Salud (RICAPPS), Avenida de Portugal 83, 37005 Salamanca, Spain; 6 Servicio de Hospitalización Domiciliaria, Hospital Universitario Marqués de Valdecilla, 39008 Santander, Spain

**Keywords:** Mediterranean diet, problem gambling, gambling-related harm, young adults, diet quality, behavioral addictions

## Abstract

**Background/Objectives:** Gambling disorder is recognized as a behavioral addiction, and gambling-related problems represent an important public health concern among young adults. However, evidence regarding their association with overall diet quality, particularly adherence to the Mediterranean diet (MedDiet), remains scarce. This exploratory secondary analysis examined whether screening indicators of gambling-related problems were associated with MedDiet adherence in young adults. **Methods:** We conducted a cross-sectional analysis of 496 participants aged 18–34 years from the EVA-Adic study. MedDiet adherence was assessed using the 14-item Mediterranean Diet Adherence Screener (MEDAS-14). Gambling-related indicators were assessed using the Short Pathological Gambling Questionnaire (CBJP), the Lie/Bet Questionnaire, and their six individual items. Independent multiple linear regression models were fitted for each indicator. The fully adjusted Model 2 included age, sex, alcohol consumption, sleep quality, sitting time, marital status, and educational level. Given the secondary nature of the analysis, the absence of an a priori sample-size calculation for the present research question, and the limited number of participants with positive gambling-related indicators, the analyses were considered exploratory and hypothesis-generating rather than confirmatory. **Results:** The mean age was 26.60 ± 4.41 years; 276 participants (55.6%) were women, and 157 (31.7%) had high MedDiet adherence. In the fully adjusted Model 2, feeling guilty about gambling (Q2) was nominally associated with a lower MEDAS-14 score (B = −1.10; 95% CI, −1.97 to −0.22; *p* = 0.015). However, this association did not remain statistically significant after Benjamini–Hochberg correction (q = 0.116), and none of the eight primary Model 2 associations met the FDR-adjusted significance threshold. **Conclusions:** Several gambling-related screening indicators showed nominal inverse associations with MedDiet adherence, with gambling-related guilt showing the most consistent estimate across sensitivity analyses. However, no association remained statistically significant in the fully adjusted model after correction for multiple testing, and formal interaction analyses provided no robust evidence of effect modification by sex. Given the small number of positive screens and the cross-sectional design, these findings should be considered exploratory and hypothesis-generating.

## 1. Introduction

The Mediterranean diet (MedDiet) is considered an optimal dietary pattern and a comprehensive lifestyle model [1,2,3]. It is characterized by a high intake of monounsaturated fats, particularly from extra-virgin olive oil, as well as fruits, vegetables, legumes, nuts, and fish, together with a low intake of red meat and ultra-processed foods, and is associated with systemic antioxidant and anti-inflammatory effects [2,3]. Adherence to the MedDiet between 18 and 35 years of age may reduce premature cardiometabolic risk and help establish healthy habits that persist over the long term [4,5]. In recent decades, contemporary sociodemographic changes have favored a shift away from this dietary pattern toward diets of lower nutritional quality and a higher prevalence of sedentary behaviors [6,7].

Gambling disorder is recognized as a behavioral addiction, and gambling-related problems constitute an important public health concern. Gambling-related harms are not restricted to people who meet clinical criteria for gambling disorder; they also include financial difficulties, family and social conflict, occupational impairment, psychological distress, and an overall reduction in well-being [8,9]. Recent epidemiological estimates indicate that gambling participation and problem gambling occur at appreciable rates in the general population, with young adults being particularly vulnerable. The prevalence of any risk gambling has been estimated at 8.7% (95% CI, 6.6–11.3), whereas the prevalence of problem gambling is approximately 1.41% (95% CI, 1.06–1.84) [9]. Male sex, younger age, financial difficulties, lower educational attainment, substance use, and mental health problems have also been identified as factors associated with a greater likelihood of problematic gambling behaviors [10].

Problem gambling rarely occurs in isolation; rather, it often coexists with broader psychological and lifestyle vulnerabilities. Recent reviews have reported substantial comorbidity with alcohol- and other substance-related disorders and with depressive and anxiety disorders [11,12]. Greater gambling severity has also been associated with several health-risk behaviors. In a recent population-based study, participants with more severe gambling problems more frequently reported low fruit and vegetable intake, lower physical activity, smoking, and obesity [13]. Although that study did not specifically evaluate MedDiet adherence, it suggests that gambling-related problems and poorer dietary habits may coexist within broader behavioral and lifestyle profiles. MedDiet adherence has also been associated with mental health and well-being outcomes [14]. The specific relationship between gambling-related problems and overall diet quality, however, has received little attention, and any observed association may reflect shared behavioral, psychological, social, socioeconomic, or health-related determinants rather than a direct effect of specific dietary components.

Epidemiological assessment of problem gambling can be performed using brief screening instruments, which are particularly useful in population-based studies. The Short Pathological Gambling Questionnaire (Cuestionario Breve de Juego Patológico, CBJP) is a tool developed and validated in the Spanish population and consists of four questions addressing self-perceived gambling problems, feelings of guilt, unsuccessful attempts to stop gambling, and the use of household money for gambling or paying gambling-related debts [15]. The questionnaire can identify participants with indicators compatible with possible gambling problems, although a positive result does not by itself constitute a clinical diagnosis. The Lie/Bet Questionnaire provides a complementary assessment using two questions focused on hiding or lying about gambling behavior and on the need to wager progressively larger amounts of money [16,17]. The brevity of both instruments facilitates their use in epidemiological studies and allows evaluation of both the overall screening result and the specific dimensions underlying problematic gambling behavior.

Despite the growing public health relevance of gambling for money and its association with several risk behaviors, the relationship between screening indicators of gambling problems and MedDiet adherence has scarcely been explored. Examining MEDAS-14 in relation to the CBJP, the Lie/Bet Questionnaire, and their individual components may provide novel information on whether gambling-related problems coexist with poorer diet quality. Studying these associations in young adults is particularly relevant because this life stage represents a period during which both eating habits and gambling-related behaviors become established and may persist into later adulthood. A previous analysis from the EVA-Adic cohort evaluated the relationship between MedDiet adherence and different digital addictive behaviors, including problematic smartphone, Internet, and video-gaming use [18]. Although both studies share the same study population and use MEDAS-14 to assess dietary adherence, the present analysis addresses a different research question, specifically focused on indicators related to gambling for money. The present study uses the CBJP, the Lie/Bet Questionnaire, and their six individual items, none of which were analyzed in the previous study. Thus, the exposures and research question evaluated here are distinct from those of the previous study and extend the investigation of behavioral profiles associated with MedDiet adherence in young adults.

The rationale for examining Mediterranean diet adherence in relation to gambling-related problems does not imply that specific components of the Mediterranean diet have a direct biological effect on gambling behavior. Rather, MEDAS-14 was considered an indicator of overall dietary pattern and diet quality. Problematic gambling and poorer dietary habits may coexist within broader behavioral and lifestyle profiles influenced by shared psychological, social, socioeconomic, and health-related determinants. Given the scarcity of previous studies directly examining this relationship, investigating the association between gambling-related screening indicators and Mediterranean diet adherence may provide preliminary evidence on whether these behaviors coexist in young adults. Therefore, the aim of the present exploratory secondary analysis was to examine the association between MedDiet adherence, assessed using MEDAS-14, and positive screening indicators derived from the CBJP, the Lie/Bet Questionnaire, and their six individual items in adults aged 18–34 years. We hypothesized that the presence of each gambling-related indicator would be associated with a lower MEDAS-14 score.

## 2. Materials and Methods

### 2.1. Study Design

The results presented in this work are part of the EVA-Adic study. The EVA-Adic study protocol has been published previously [19]. EVA-Adic is a cross-sectional observational study conducted at the Primary Care Research Unit of Salamanca (APISAL). The study was registered at ClinicalTrials.gov (NCT05866133) and was first posted on 19 May 2023.

### 2.2. Study Population

The EVA-Adic protocol initially planned age- and sex-stratified random sampling using the health-card database as the sampling frame. However, access to this database was ultimately unavailable, making the planned probabilistic sampling strategy unfeasible. Recruitment was therefore conducted through an active, open strategy using different dissemination channels targeting young adults in the study area. The main inclusion and exclusion criteria were included in the recruitment information, and interested individuals were subsequently screened by the research team to confirm eligibility before consecutive enrollment.

Between 17 March 2023 and 29 January 2025, 501 participants aged 18–34 years residing in the urban health area of Salamanca were recruited using this open, consecutive, non-probabilistic strategy. Inclusion criteria were age 18–34 years and provision of written informed consent; exclusion criteria were terminal illness and inability to attend the research unit for study assessments. Participants completed the study procedures during two visits to the research center, with all assessments performed within an interval of less than eight days. Of the 501 recruited participants, 496 (99.0%; 220 men and 276 women) had complete information required for the present analyses. Because recruitment was based on open dissemination rather than individual invitations from a predefined sampling frame, the total number of individuals exposed to the recruitment information, those who chose not to respond, and consequently a conventional participation rate could not be determined.

**Figure 1:**
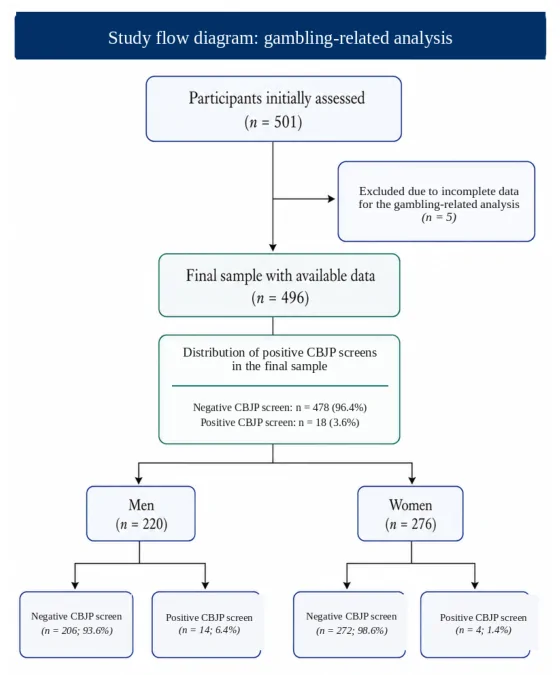
Flow diagram of participants included in the gambling-related analysis. Among the 501 initially assessed participants, 5 were excluded because of incomplete data, resulting in a final analytical sample of 496 participants. Overall, 18 participants (3.6%) had a positive CBJP screen for gambling-related problems, including 14 men (6.4%) and 4 women (1.4%).

The inclusion of participants in the present analysis is summarized in the study flow diagram (Figure 1).

### 2.3. Study Size

The sample size of the EVA-Adic study was established previously according to the primary objective described in the published protocol [19]. The present work is a secondary analysis of that cohort, and no specific a priori sample-size calculation was performed for the association between gambling-related indicators and MedDiet adherence. All participants with complete information for all variables analyzed were included. Given the secondary nature of the analysis, the absence of an a priori sample-size calculation for the present research question, and the limited number of participants with positive gambling-related indicators, the analyses were considered exploratory and hypothesis-generating rather than confirmatory.

The study followed the Strengthening the Reporting of Observational Studies in Epidemiology (STROBE) guidelines throughout all stages [20]; the corresponding checklist is provided in Supplementary Table S1.

### 2.4. Variables and Measurement Instruments

The questionnaires used in the study were collected within a period of less than 8 days. Assessments were performed in a standardized manner by three previously trained investigators, and quality was evaluated by an independent investigator. All assessments were conducted under standardized environmental conditions, and the questionnaires were administered sequentially.

#### 2.4.1. Sociodemographic Variables and Personal History

Age, sex, educational level, and marital status were recorded at study inclusion using a structured clinical interview.

#### 2.4.2. Lifestyle

Alcohol consumption was assessed using a structured self-administered questionnaire that recorded the amount and type of alcohol habitually consumed over one week. Alcohol intake was expressed in grams per week. Sedentary behavior was assessed using the Marshall Sitting Questionnaire (MSQ), a self-administered questionnaire that estimates sitting time across different contexts of daily life and has been validated in the Spanish population [21]. Participants separately reported, for a typical weekday and a typical weekend day, the daily time spent sitting in five domains: travel or transport, work or class attendance, television viewing, home computer use, and other seated leisure activities excluding television viewing. Total weekly sitting time was calculated by summing the daily minutes across the five domains over seven days using the following formula: total weekly sitting time = 5 × weekday + 2 × weekend day. The result was converted to hours per week and analyzed as a continuous quantitative variable, with higher values indicating greater weekly sedentary time.

#### 2.4.3. Mediterranean Diet

MedDiet adherence was assessed using the 14-item Mediterranean Diet Adherence Screener (MEDAS-14) [22], originally developed within the PREDIMED (PREvención con DIeta MEDiterránea) study [23] and validated in older Spanish adults at high cardiovascular risk [22]. The self-administered questionnaire consists of 14 closed-ended items assessing the consumption of key foods and characteristic behaviors of the Mediterranean dietary pattern. The overall score ranges from 0 to 14 points, with higher scores indicating greater adherence. The ≥9-point threshold derives from the PREDIMED setting [22,23].

Following the standardized methodological criteria used in the PREDIMED cohort [23,24], participants were classified as having low-to-moderate adherence when the MEDAS-14 score was <9 and high adherence when the score was ≥9. Subsequent cross-national research has supported the validity of MEDAS-14 in broader adult populations, including Spanish participants [25]. Nevertheless, specific validation data for Spanish adults aged 18–34 years remain limited. Therefore, MEDAS-14 was analyzed primarily as a continuous score, whereas the ≥9-point cut-off was used as a complementary categorical measure.

#### 2.4.4. Assessment of Gambling-Related Variables

Gambling-for-money behaviors were assessed using the Short Pathological Gambling Questionnaire (Cuestionario Breve de Juego Patológico, CBJP) and the Lie/Bet Questionnaire, both of which are screening rather than diagnostic instruments. The CBJP was developed and validated in the Spanish population from four items selected from the Spanish version of the South Oaks Gambling Screen [15]. Its items assess self-perceived gambling problems, feelings of guilt, unsuccessful attempts to stop gambling, and the use of household money to gamble or pay gambling-related debts. Each affirmative response was scored as 1 point and each negative response as 0 points, yielding a total score from 0 to 4; according to the validation criterion, a score ≥ 2 was considered a positive screen for gambling-related problems. The Lie/Bet Questionnaire comprises two items addressing the need to gamble increasing amounts of money (“Bet”) and lying to significant others about gambling behavior (“Lie”) [16]. Each response was coded as 0 = no and 1 = yes, yielding a score from 0 to 2. Consistent with the original scoring criterion and subsequent population-based validation, a positive screen was defined as at least one affirmative response (score ≥ 1) [16,17]. The published EVA-Adic protocol incorrectly stated that two affirmative responses were required; this was a reporting error in the protocol manuscript rather than a change in the scoring criterion or analytical strategy used in the present study. In addition to the two overall screening results, the six component items were analyzed individually as dichotomous variables coded 0 = absence and 1 = presence: Q1, self-perceived gambling problems; Q2, feeling guilty about gambling; Q3, unsuccessful attempt to stop gambling; Q4, use of household money to gamble or pay debts; Q5, having lied to important people about gambling behavior; and Q6, the need to bet increasing amounts of money. The overall CBJP screen, the overall Lie/Bet screen, and each individual item were entered into separate statistical models, avoiding simultaneous inclusion of composite screens and the items from which they were derived.

### 2.5. Statistical Analysis

Statistical analyses were performed using IBM SPSS Statistics for Windows, version 30.0 (IBM Corp., Armonk, NY, USA). All tests were two-sided. The analytical sample comprised 496 participants with complete information for MEDAS-14, the gambling-related indicators, and all covariates used in the main regression models; five participants with incomplete data were excluded. Quantitative variables are presented as mean ± standard deviation (SD), and categorical variables as n (%). Graphical displays of MEDAS-14 scores show mean ± standard error (SE). Participant characteristics were examined in the overall sample and compared by sex and by MedDiet adherence category (<9 vs. ≥9 MEDAS-14 points). Continuous variables were compared using Student’s *t*-test or Welch’s *t*-test as appropriate, and categorical variables using Pearson’s chi-square test or Fisher’s exact test when expected cell counts were <5. These descriptive comparisons were interpreted using nominal *p* values. MEDAS-14 score was the primary dependent variable and was analyzed continuously. The eight dichotomous exposures were the positive CBJP screen, the positive Lie/Bet screen, and Q1–Q6. Each gambling-related indicator was entered separately into an independent linear regression model. A minimally adjusted model included age and sex. Model 1 additionally included alcohol consumption (g/week), Pittsburgh Sleep Quality Index score, and sitting time. Because these lifestyle factors may act as confounders or may lie partly on pathways linking gambling-related problems with dietary behavior, differences across sequential models were examined to assess the impact of their inclusion. Model 2 additionally included marital status and educational level. Given the cross-sectional design, these models were interpreted as adjusted associations rather than causal effect estimates. Results are reported as unstandardized regression coefficients (B), 95% confidence intervals (95% CI), and *p* values. Model-specific R^2^ values were also calculated.

A hierarchical analytical framework was used to address multiplicity. The global CBJP positive screen was designated as the primary gambling-related exposure, the global Lie/Bet screen as a secondary exposure, and the six individual items as exploratory exposures. The fully adjusted Model 2 was considered the primary inferential model; the eight Model 2 associations constituted the primary family of tests and were adjusted using the Benjamini–Hochberg false discovery rate (FDR) procedure. The eight Model 1 associations were evaluated as a separate supportive FDR family. The minimally adjusted model was used to assess consistency across progressive adjustment but was not included in the primary multiplicity-controlled family. FDR-adjusted *p* values are reported as q values, with q < 0.05 considered statistically significant after correction. Multicollinearity in Models 1 and 2 was assessed using tolerance and the variance inflation factor (VIF), with VIF < 2 considered indicative of low collinearity. Potential effect modification by sex was evaluated in the pooled sample by adding the corresponding gambling indicator × sex interaction term, together with both main effects, to Model 1 and Model 2. Sex was coded as 0 = man and 1 = woman, and each gambling-related indicator as 0 = absence and 1 = presence. The seven estimable interaction terms within each model were treated as separate supportive FDR families. The interaction for Q4 could not be estimated because no women endorsed this indicator. Sex-stratified analyses were considered exploratory and were not used as evidence of effect modification. Given the small number of participants endorsing some indicators, additional robustness analyses were performed for the fully adjusted model using 5000 bootstrap resamples with bias-corrected and accelerated (BCa) 95% confidence intervals when estimable. Potentially influential observations were assessed using studentized deleted residuals, Cook’s distance, and leverage. Leave-one-out sensitivity analyses were also performed by sequentially excluding each participant endorsing the corresponding gambling indicator and refitting the fully adjusted model.

To address potential mathematical coupling between alcohol consumption as a covariate and the wine component of MEDAS-14, a sensitivity analysis was conducted using a modified 13-item score (MEDAS-13), calculated by subtracting the wine item from MEDAS-14. The fully adjusted models were repeated using MEDAS-13 as the dependent variable while retaining total alcohol intake as a covariate. As an additional sensitivity analysis, the original MEDAS-14 models were repeated after excluding alcohol intake from the covariate set. To further evaluate potential residual confounding, an extended covariate sensitivity model was fitted using MEDAS-13 and additionally adjusting for smoking status, physical activity, body mass index, employment status, and problematic smartphone, Internet, and videogame use assessed with the EDAS-18, CIUS, and CERV scores, respectively. This extended model was considered a sensitivity analysis and not part of the primary inferential framework.

### 2.6. Ethical Considerations

The Research Ethics Committee for Medicinal Products of the Salamanca Health Area approved the first phase of the project on 10 July 2021 (CEIm reference code PI 2021 088671048) and the second phase on 24 July 2023 (CEIm reference code PI 2023 071332). The study was conducted in accordance with the Declaration of Helsinki [26]. All participants provided written informed consent before inclusion after receiving detailed information about the study procedures.

### 2.7. Use of Artificial Intelligence Tools

During manuscript preparation, the authors used ChatGPT (OpenAI, San Francisco, CA, USA; version 5.5, accessed on 30 July 2026) to improve clarity in English and to assist with figure design based exclusively on statistical results generated by the authors. The tool was not used to generate data, perform statistical analyses, or interpret findings. All results were critically reviewed and verified by the authors, who assume full responsibility for the final content.

## 3. Results

Table 1 presents the baseline characteristics of the overall population and stratified by sex. The sample included 496 participants, of whom 220 were men and 276 were women. Women were younger, had a higher educational level, and had higher MedDiet scores and adherence than men. Regarding lifestyle factors, men reported higher alcohol intake. Gambling-related variables were infrequent in the overall sample. Nevertheless, gambling problems assessed using the CBJP, suspected gambling problems according to the Lie/Bet Questionnaire, and affirmative responses to Q1 (self-perceived gambling problems), Q2 (feeling guilty about gambling), Q4 (taking household money to gamble or pay debts), and Q6 (need to bet increasing amounts of money) were more frequent among men. No significant sex differences were observed for Q3 or Q5.

Table 2 presents baseline characteristics according to the degree of MedDiet adherence. Participants with high adherence were older on average and had a higher proportion of university education than those with low-to-moderate adherence. Gambling-related variables were infrequent in both groups. The proportion of participants reporting guilt about gambling (Q2) was significantly higher in the low-to-moderate adherence group than in the high-adherence group (5.6% vs. 1.3%; *p* = 0.026). No significant differences were observed for a positive CBJP screen, suspected gambling problems according to the Lie/Bet Questionnaire, or the remaining gambling-related items. Notably, Q2 was the only gambling-related indicator showing a nominal between-group difference when MedDiet adherence was categorized and also the only indicator retaining a nominal association in the fully adjusted model using the continuous MEDAS-14 score. The broader pattern observed with continuous MEDAS-14 scores should therefore be interpreted in light of the different outcome parameterization and the loss of information inherent in dichotomization.

**Figure 2:**
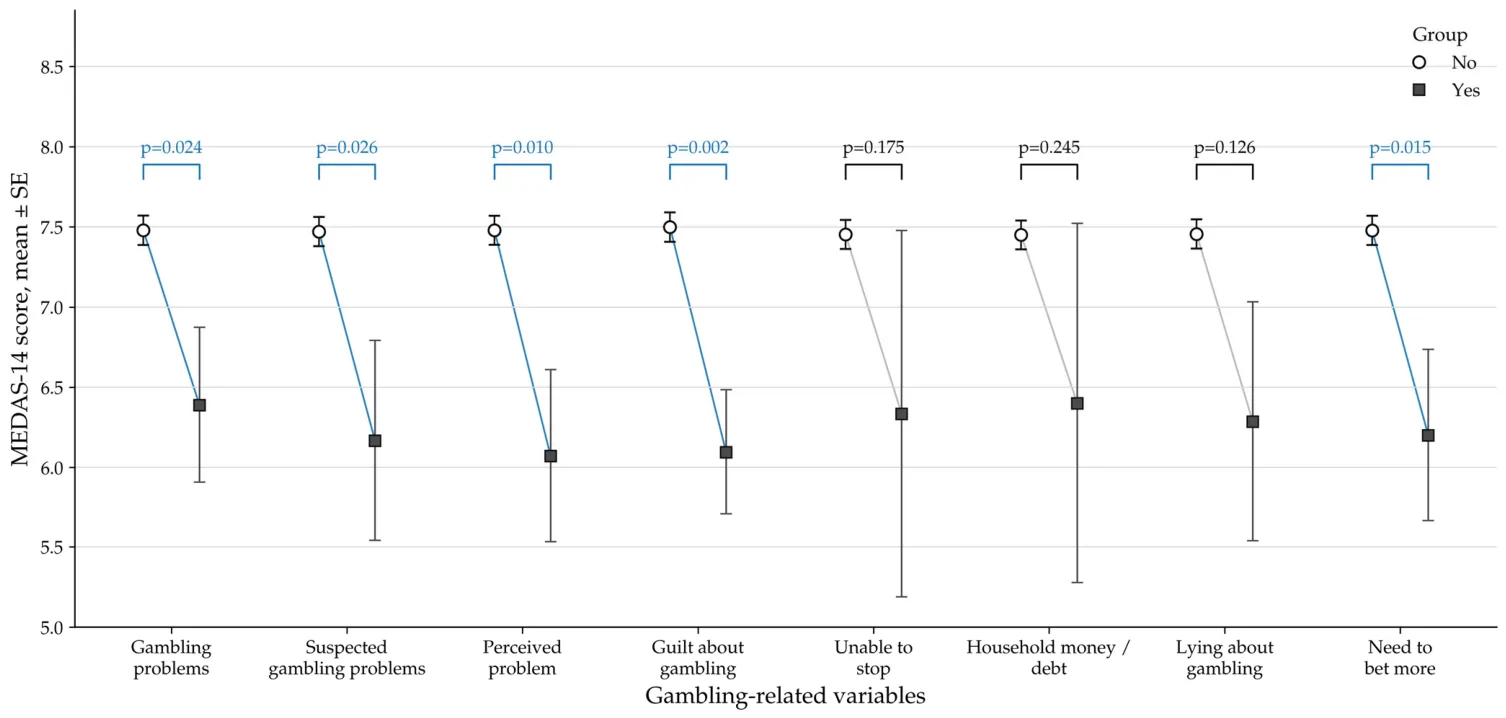
Mediterranean diet adherence according to gambling-related screening indicators. MEDAS-14 scores are shown as mean ± standard error according to the presence or absence of each indicator. Blue annotations denote nominal between-group differences with *p* < 0.05; these descriptive comparisons were not corrected for multiple testing. Abbreviations: MEDAS-14, 14-item Mediterranean Diet Adherence Screener; SE, standard error.

Figure 2 shows mean MEDAS-14 scores according to the presence or absence of each gambling-related screening indicator. Participants with positive indicators generally had lower mean MedDiet adherence scores than those without the corresponding indicators. Nominal between-group differences (*p* < 0.05) were observed for the positive CBJP screen, the positive Lie/Bet screen, Q1, Q2, and Q6.

**Figure 3:**
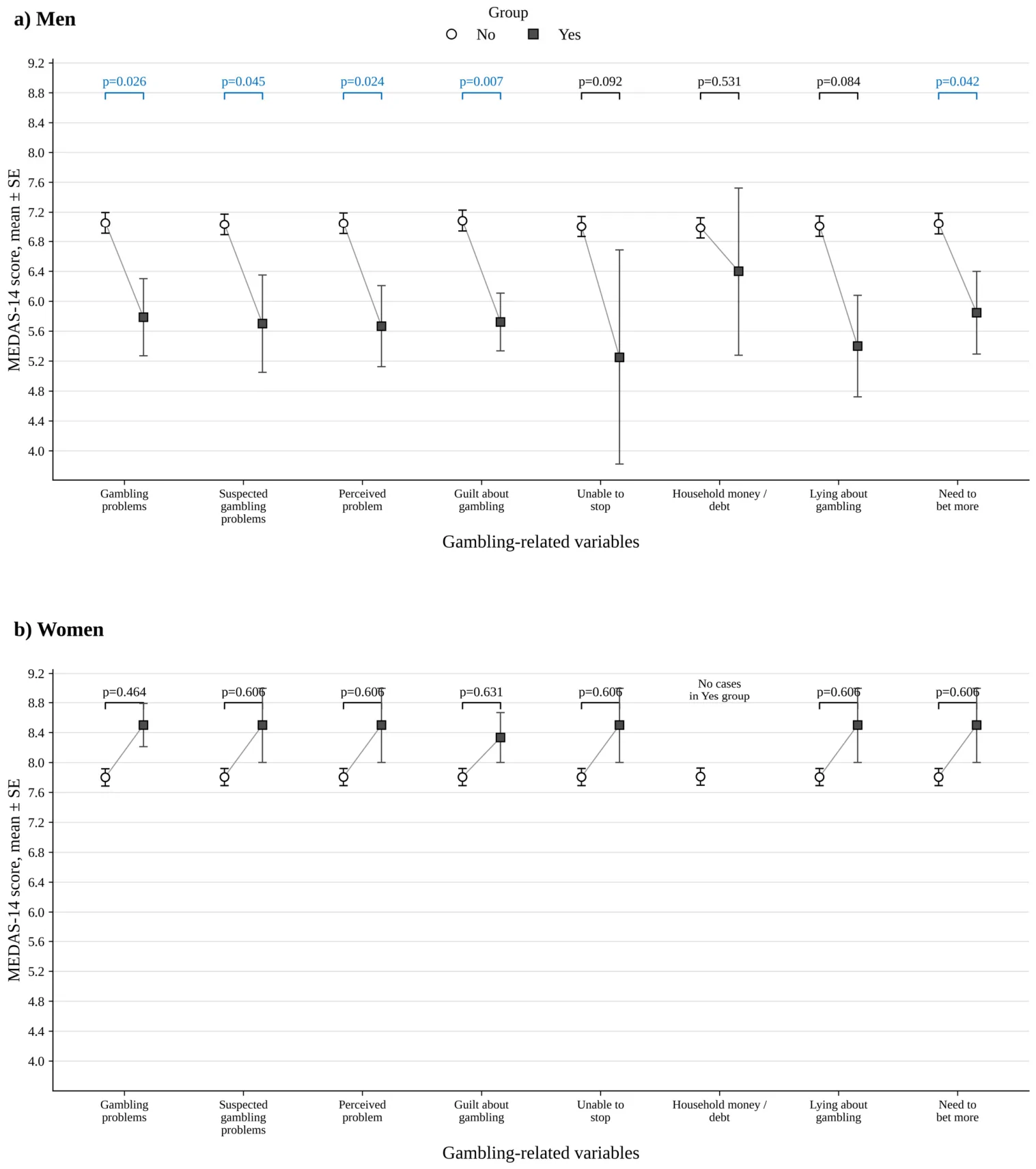
Exploratory Mediterranean diet adherence comparisons according to gambling-related screening indicators, stratified by sex. MEDAS-14 scores are shown as mean ± standard error in men (**a**) and women (**b**). Blue annotations denote nominal *p* < 0.05. Positive counts among women were: CBJP, n = 4; Lie/Bet, n = 2; Q1, n = 2; Q2, n = 3; Q3, n = 2; Q4, n = 0; Q5, n = 2; and Q6, n = 2. These descriptive comparisons were not corrected for multiple testing and should not be interpreted as evidence of sex-specific effect modification. Abbreviations: CBJP, Cuestionario Breve de Juego Patológico; MEDAS-14, 14-item Mediterranean Diet Adherence Screener; Q, question; SE, standard error.

Exploratory sex-stratified descriptive comparisons are shown in Figure 3. Among men, nominal between-group differences were observed for the positive CBJP screen, the positive Lie/Bet screen, Q1, Q2, and Q6. No nominal differences were observed among women. Because the number of positive screens among women was very small, these within-sex comparisons were considered descriptive and were not interpreted as evidence of effect modification.

Supplementary Table S2 presents the collinearity diagnostics for the multiple linear regression models. Across all predictors, tolerance values ranged from 0.923 to 0.975 in Model 1 and from 0.657 to 0.975 in Model 2, whereas VIF values ranged from 1.026 to 1.084 and from 1.026 to 1.523, respectively. All VIF values were <2, indicating no relevant multicollinearity. In the minimally adjusted models including age and sex, all gambling-related estimates were negative, with nominal inverse associations for Q1, Q2, and Q6. Further adjustment for alcohol consumption, sleep quality, and sitting time did not systematically attenuate the coefficients, which remained broadly similar in magnitude.

**Figure 4:**
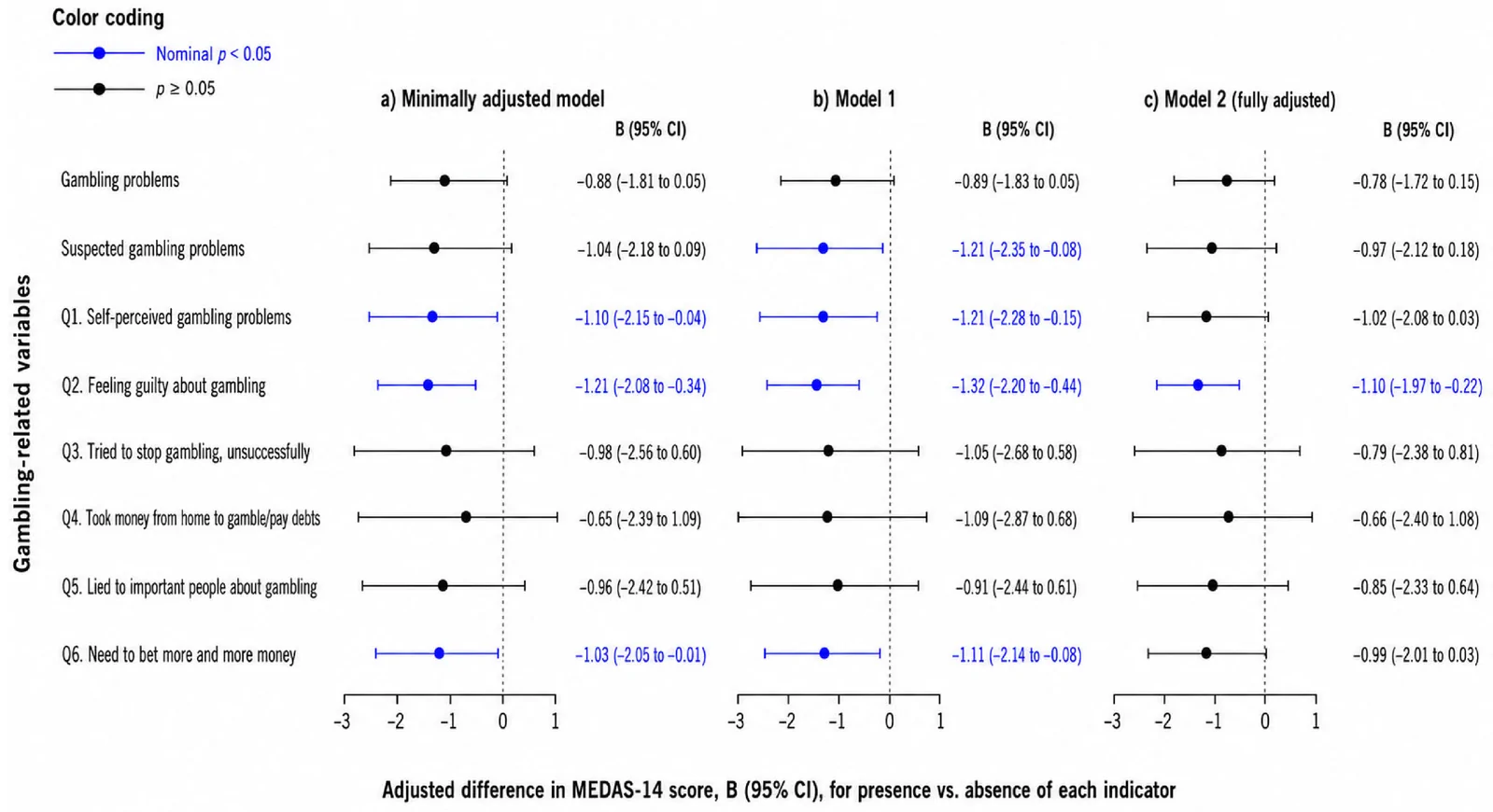
Associations between gambling-related indicators and Mediterranean diet adherence across sequentially adjusted linear regression models. Forest plots show unstandardized regression coefficients (B) and 95% confidence intervals (95% CI), representing the adjusted difference in MEDAS-14 score between participants with versus without each gambling-related indicator. (**a**) The minimally adjusted model included age and sex. (**b**) Model 1 additionally included alcohol consumption (g/week), Pittsburgh Sleep Quality Index score, and sitting time. (**c**) Model 2 (fully adjusted) additionally included marital status and educational level. Each gambling-related indicator was examined in a separate regression model. Negative coefficients indicate lower MEDAS-14 scores among participants with the corresponding indicator. The vertical dashed line denotes the null value (B = 0). Blue estimates indicate nominal *p* < 0.05, whereas black estimates indicate *p* ≥ 0.05. In Model 2, only Q2 remained nominally associated with a lower MEDAS-14 score (B = −1.10; 95% CI, −1.97 to −0.22), but this association did not survive Benjamini–Hochberg FDR correction (q = 0.116); none of the Model 2 associations met the FDR-adjusted significance threshold. Abbreviations: B, unstandardized regression coefficient; CI, confidence interval; FDR, false discovery rate; MEDAS-14, 14-item Mediterranean Diet Adherence Screener; Q, question.

Figure 4 summarizes the sequential regression models. In the minimally adjusted model, adjusted only for age and sex, all gambling-related coefficients were negative, with nominal inverse associations for Q1, Q2, and Q6. After further adjustment in Model 1 for alcohol consumption, sleep quality, and sitting time, nominal inverse associations were observed for the positive Lie/Bet screen, Q1, Q2, and Q6, whereas the positive CBJP screen did not reach nominal statistical significance. After Benjamini–Hochberg FDR correction across the eight Model 1 tests, only the association with gambling-related guilt (Q2) remained statistically significant (q = 0.038). In the fully adjusted Model 2, estimates were further attenuated, and only Q2 remained nominally associated with a lower MEDAS-14 score (B = −1.10; 95% CI, −1.97 to −0.22; *p* = 0.015). However, this association did not remain statistically significant after FDR correction (q = 0.116), and none of the eight primary Model 2 associations met the FDR-adjusted significance threshold. Accordingly, the fully adjusted item-level findings were interpreted as exploratory and hypothesis-generating rather than confirmatory.

Formal sex-interaction analyses provided no robust evidence of effect modification by sex (Supplementary Table S3). In Model 1, none of the seven estimable interaction terms reached nominal statistical significance and none survived FDR correction (all q ≥ 0.172). In the fully adjusted Model 2, nominal interactions were observed for the positive CBJP screen (*p* = 0.042) and Q2 (*p* = 0.044); however, neither remained statistically significant after Benjamini–Hochberg FDR correction (both q = 0.101), and no other estimable interaction survived correction. The Q4 interaction could not be estimated because no women endorsed this indicator.

**Figure 5:**
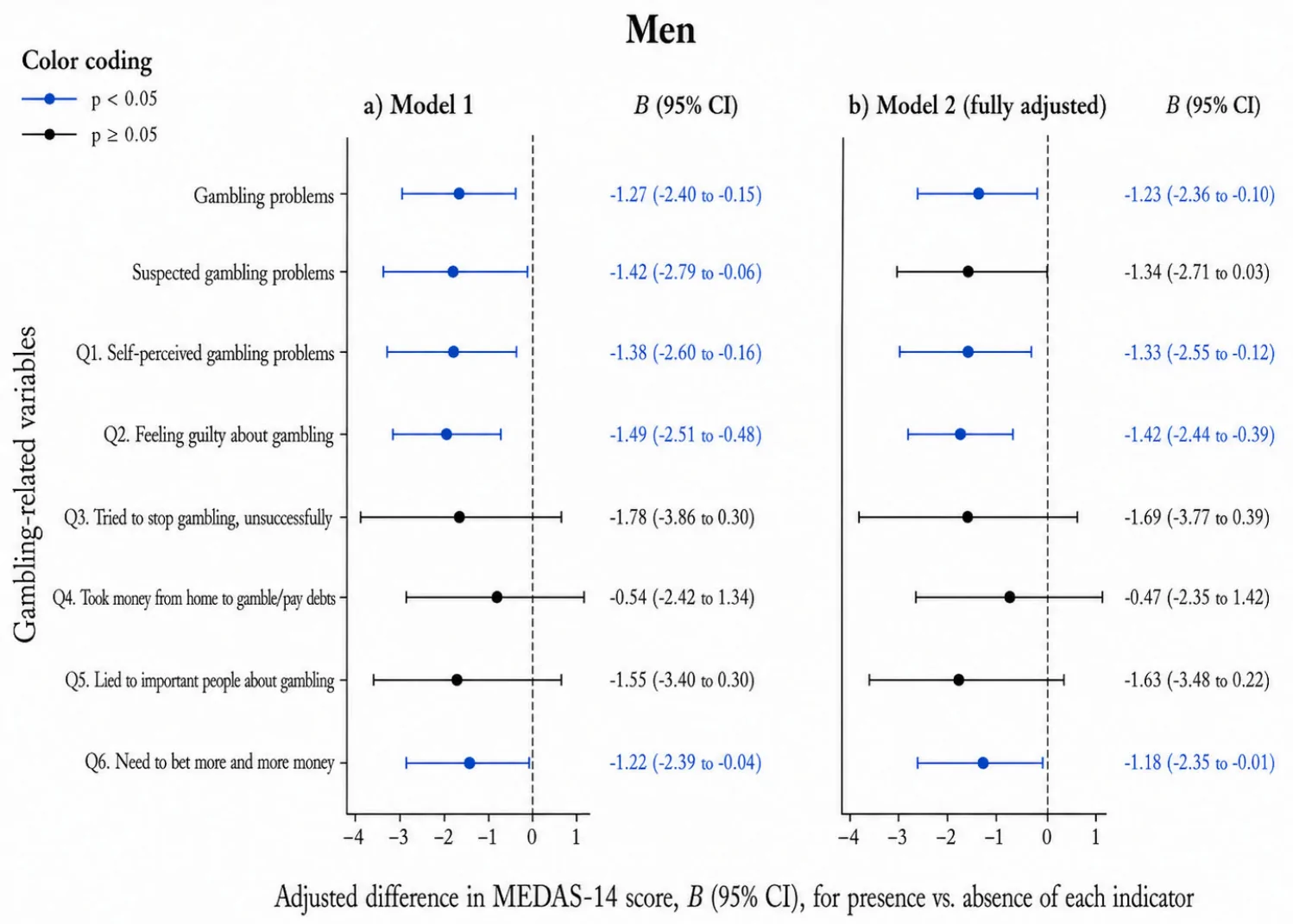
Exploratory sex-stratified associations between gambling-related indicators and Mediterranean diet adherence among men. Unstandardized multiple linear regression coefficients (B) and 95% confidence intervals are shown for each indicator analyzed separately. Model 1 was adjusted for age, alcohol consumption, Pittsburgh Sleep Quality Index score, and sitting time; Model 2 additionally included marital status and educational level. The dashed vertical line represents the null value (B = 0). Blue estimates indicate nominal *p* < 0.05 and black estimates *p* ≥ 0.05. These within-men estimates should not be interpreted as evidence of sex-specific effect modification; formal interaction tests are reported in Supplementary Table S3. Abbreviations: B, unstandardized regression coefficient; CI, confidence interval; MEDAS-14, 14-item Mediterranean Diet Adherence Screener.

Exploratory sex-stratified regression estimates are shown in Figure 5 and Supplementary Table S4. Among men, negative estimates reached nominal *p* < 0.05 for the positive CBJP screen, Q1, Q2, and Q6 in both models; the positive Lie/Bet screen reached nominal significance only in Model 1. Among women, no association reached nominal significance and confidence intervals were wide because of the very small number of positive screens; Q4 could not be estimated because no women endorsed this indicator. These within-sex estimates were not interpreted as evidence of effect modification, which was assessed using formal interaction terms in the pooled sample.

Cross-tabulation showed substantial overlap among gambling-related indicators; eight participants screened positive on both composite measures, with frequent co-occurrence between the composite screens and individual items (Supplementary Table S5).

The proportion of variance in MEDAS-14 explained by the regression models was modest; model-specific R^2^ values for the minimally adjusted model, Model 1, and Model 2 are provided in Supplementary Table S6.

Bootstrap and leave-one-out sensitivity analyses showed heterogeneous robustness across gambling indicators. Gambling-related guilt showed the most stable inverse association, remaining negative and nominally significant in all 21 leave-one-out iterations (B range, −1.235 to −0.940). Q1 also remained negative across all 14 iterations (B range, −1.249 to −0.790), although nominal statistical significance was sensitive to individual exclusions. Indicators with fewer positive participants showed greater instability. Influence diagnostics identified only one participant with an absolute studentized deleted residual >3, and the maximum Cook’s distance was ≤0.180; no observation showed evidence of extreme influence.

Excluding the wine component from MEDAS-14 had virtually no effect on the estimates. Only one participant met the wine-item criterion, and results obtained using MEDAS-13 were essentially unchanged. For gambling-related guilt, the fully adjusted association was B = −1.095 (95% CI, −1.974 to −0.215; *p* = 0.015). Similarly, excluding alcohol intake from the covariate set while retaining MEDAS-14 produced a comparable estimate (B = −1.110; 95% CI, −1.987 to −0.232; *p* = 0.013). In the extended sensitivity model additionally accounting for smoking, physical activity, body mass index, employment status, and problematic smartphone, Internet, and videogame use, the estimate for gambling-related guilt remained similar in magnitude (B = −1.035; 95% CI, −1.944 to −0.125; *p* = 0.026). No relevant multicollinearity was observed (maximum VIF approximately 2.05). However, Q2 did not remain statistically significant after FDR correction in this extended model (q = 0.207), and no association in the extended sensitivity analysis survived multiplicity correction.

## 4. Discussion

In this cross-sectional study of young adults, positive screening indicators for gambling-related problems tended to coexist with lower Mediterranean diet adherence scores. However, the strength and robustness of these associations varied across indicators, and none of the eight gambling-related associations remained statistically significant in the fully adjusted Model 2 after Benjamini–Hochberg FDR correction. The individual gambling items represent overlapping components of the CBJP and Lie/Bet screening instruments and therefore should not be regarded as independent confirmatory tests. Gambling-related guilt showed the most consistent inverse estimate across bootstrap, leave-one-out, modified MEDAS, and extended covariate sensitivity analyses; nevertheless, this association did not survive FDR correction after full covariate adjustment. Formal interaction analyses likewise provided no robust evidence of effect modification by sex after correction for multiple testing. Accordingly, the findings should be regarded as exploratory and hypothesis-generating rather than confirmatory. Furthermore, the cross-sectional design precludes inference regarding the temporal or causal direction of these associations.

The 3.6% frequency of positive CBJP screens observed in EVA-Adic was somewhat higher than several estimates cited for problem or pathological gambling in young populations. An international systematic review and meta-analysis estimated that 8.7% of adults engaged in some form of risk gambling and that 1.41% met criteria for problem gambling [9]. Among Spanish young adults aged 18–25 years, López-del-Hoyo et al. [27] identified 2.4% of university students with a screen compatible with possible pathological gambling, whereas Krotter et al. [28] reported frequencies of 3.51%, 2.14%, and 0.51% for low-risk, moderate-risk, and problem gambling, respectively. Direct comparisons should be made cautiously because the EVA-Adic estimate reflects a positive screening result rather than a clinical diagnosis, whereas several previous estimates were based on diagnostic or severity-based classifications, and instruments, cut-off points, assessment periods, and sample characteristics differed across studies. Accordingly, the CBJP and Lie/Bet results in EVA-Adic should be interpreted as identifying possible gambling-related problems rather than establishing the prevalence of clinically diagnosed gambling disorder.

The proportion of participants with high MedDiet adherence in EVA-Adic was 31.7%, close to the 29.3% recently reported in a sample of Spanish university students [29]. In both studies, women showed greater adherence to the Mediterranean dietary pattern. Positive gambling-related screens and items were also more frequent among men in EVA-Adic, consistent with international epidemiological evidence and studies among young Spanish people showing greater vulnerability to risk or problem gambling among men [9,10,28,30]. However, these descriptive sex differences should not be conflated with sex-specific associations between gambling-related indicators and MedDiet adherence. Formal interaction analyses provided no robust evidence of effect modification by sex after FDR correction, and the very small number of positive screens among women limited the precision of stratified estimates.

Direct evidence examining the relationship between gambling-related problems and overall diet quality remains scarce. In a Finnish population-based study, daily smoking, heavy alcohol consumption, low fruit and vegetable intake, and insufficient sleep became more frequent as gambling severity increased [13]. These patterns were not confined to participants with the most severe problems but were also observed among those at low or moderate risk. EVA-Adic adds exploratory evidence by examining an overall dietary pattern using MEDAS-14 rather than isolated eating behaviors, while the cross-sectional design prevents conclusions about directionality or causation.

The findings can also be contextualized by studies examining the Mediterranean diet and psychosocial health. Among Spanish university students, greater adherence has been associated with more favorable lifestyle habits and better mental and emotional well-being [29]. In Spanish adolescents, greater adherence was associated with fewer psychosocial and behavioral difficulties [31], whereas among Greek university students it was associated with lower perceived stress and better sleep quality [32]. These studies did not evaluate gambling behaviors and therefore do not directly confirm the EVA-Adic findings. Rather than indicating a biological effect of specific Mediterranean diet components, the observed pattern may reflect shared behavioral and contextual determinants. Sleep disturbances, substance use, lower physical activity, psychological stress, disruption of daily routines, and psychosocial circumstances could influence both gambling-related behaviors and food choices. Socioeconomic circumstances may also be shared determinants. In particular, financial strain or reduced disposable income could affect meal planning and the purchase and preparation of fresh foods while also being associated with gambling-related problems. However, household income, disposable income, and financial strain were not measured. These possibilities were not directly assessed and should be regarded as hypotheses for future longitudinal research rather than demonstrated mechanisms.

The magnitude of the observed differences should also be interpreted cautiously. Although some gambling-related indicators were associated with approximately one-point-lower MEDAS-14 scores in nominal analyses, the clinical relevance of a difference of this magnitude has not been established. Therefore, these estimates should be interpreted as differences in Mediterranean diet adherence scores rather than as clinically meaningful changes in dietary status.

Given the exploratory nature of the findings and the absence of associations surviving FDR correction in the fully adjusted model, the present results do not support using dietary habits to identify individuals who should undergo gambling screening, nor do they support Mediterranean diet promotion as a specific strategy for preventing or treating gambling-related problems. Assessment of gambling-related problems in clinical practice should continue to be guided by established clinical indications and validated screening approaches rather than by dietary characteristics. Available evidence indicates that brief gambling screening instruments may be feasible and acceptable in healthcare settings [33,34], but the present study does not establish that poorer diet quality should itself trigger such screening. Conversely, although assessment of general health behaviors may form part of a comprehensive evaluation of individuals with gambling-related problems, the EVA-Adic findings do not demonstrate a specific clinical role for assessing Mediterranean diet adherence in this context. Future longitudinal studies should determine whether dietary quality and gambling-related problems share common behavioral, psychosocial, or socioeconomic determinants and whether their co-occurrence has practical relevance in clinical or public health settings.

Strengths of the study include the use of validated gambling screening instruments, standardized assessment procedures, and the high proportion of participants with complete data. MedDiet adherence was analyzed primarily as a continuous score, with the ≥9-point threshold used only as a complementary categorical measure. Each gambling-related indicator was examined in a separate model, the impact of progressive covariate adjustment was made explicit, multiplicity was addressed using an explicit analytical hierarchy and FDR correction, and additional bootstrap, influence, leave-one-out, modified-MEDAS, and extended-covariate sensitivity analyses were performed. The overlap between composite screens and individual items was also quantified to avoid interpreting correlated indicators as independent evidence.

This study nevertheless has several limitations. First, the recruitment strategy deviated from the probabilistic sampling procedure initially planned in the EVA-Adic protocol. Because access to the health-card-based sampling frame was unavailable, an active, consecutive, non-probabilistic strategy was used in a single urban area. This may have introduced selection and self-selection bias, limits representativeness and generalizability, and precluded calculation of a conventional participation rate. Second, the cross-sectional design precludes determination of temporal sequence and causal inference. Lower MedDiet adherence could precede gambling-related problems, arise alongside them, or reflect shared determinants. In addition, alcohol consumption, sleep quality, and sitting time could function as confounders or lie partly on a causal pathway; the sequential models make the impact of their inclusion transparent but cannot resolve their causal role. Third, MEDAS-14 was originally validated in older adults at high cardiovascular risk, and the ≥9-point cut-off derives from PREDIMED. Although broader adult validation data are available, its measurement properties and categorical threshold have not been specifically established in Spanish adults aged 18–34 years. Fourth, the number of positive gambling screens was small, particularly among women and for several individual items. This limited statistical power, widened confidence intervals, and increased the potential for sparse-data instability. Bootstrap, influence diagnostics, and leave-one-out analyses supported the stability of some estimates, particularly that for gambling-related guilt, but they cannot overcome the limited information in sparse exposure groups. Sex-stratified analyses should therefore be considered exploratory, and formal interaction analyses provided no robust evidence of effect modification by sex after multiplicity correction; the Q4 interaction could not be estimated because no women endorsed that item. Fifth, although FDR correction was applied to the primary Model 2 family and separately to supportive Model 1 and interaction families, the study involved multiple correlated analyses of overlapping screening indicators. Residual risk of chance findings therefore remains, and item-level results should not be interpreted as independent confirmatory evidence. Sixth, diet, gambling, sleep, alcohol consumption, and sitting time were assessed using self-reported questionnaires and are susceptible to recall and social-desirability bias, particularly for a stigmatized behavior such as gambling. The CBJP and Lie/Bet are screening instruments rather than diagnostic assessments, and individual items should not be interpreted as diagnoses. Seventh, detailed information on gambling frequency, duration, amount wagered, online versus in-person modality, gambling type, age at onset, and functional consequences was unavailable. Eighth, household income, disposable income, and financial strain were not assessed. Educational level and employment status in sensitivity analyses are only partial proxies for socioeconomic position; residual confounding by financial resources may therefore partly account for the observed associations. Finally, sex was recorded in binary form, and gender-related factors that may influence gambling patterns, problem expression, or willingness to disclose them were not assessed. Larger longitudinal and multicenter studies with greater representation across levels of gambling severity and sufficient numbers of women are needed to confirm these signals and clarify their temporal, behavioral, psychological, and socioeconomic context.

## 5. Conclusions

In this exploratory secondary analysis of young adults, several gambling-related screening indicators showed nominal inverse associations with Mediterranean diet adherence, with gambling-related guilt showing the most consistent estimate across sensitivity analyses. However, none of the eight primary associations in the fully adjusted Model 2 remained statistically significant after Benjamini–Hochberg false discovery rate correction, and formal interaction analyses provided no robust evidence of effect modification by sex. The small number of positive gambling screens resulted in imprecise estimates, and the cross-sectional design precludes inference regarding temporality or causality. Accordingly, these findings should be regarded as preliminary and hypothesis-generating rather than confirmatory. Larger prospective studies specifically designed to investigate this relationship are needed to confirm these signals, establish temporality, and clarify potential sex differences.

## Figures and Tables

**Table 1:** Baseline characteristics of the study population overall and by sex.

Variable	Overall (n = 496)	Men (n = 220)	Women (n = 276)	*p*-Value
Sociodemographic characteristics				
Age, years	26.60 ± 4.41	27.09 ± 4.40	26.22 ± 4.38	0.029
Marital status, n (%)				0.944
Married or partnered	94 (19.0)	42 (19.1)	52 (18.8)	
Not married or not partnered	402 (81.0)	178 (80.9)	224 (81.2)	
Educational level, n (%)				0.012
University, n (%)	301 (60.7)	120 (54.5)	181 (65.6)	
Non-university studies	195 (39.3)	100 (45.5)	95 (34.4)	
Lifestyle				
Alcohol intake, g/week	48.93 ± 73.90	61.56 ± 91.54	38.86 ± 54.11	0.001
Sitting time, hours/week	54.85 ± 19.78	54.24 ± 20.33	55.34 ± 19.36	0.537
Pittsburgh Sleep Quality Index score, points	5.95 ± 3.03	5.70 ± 2.92	6.16 ± 3.11	0.097
Mediterranean diet adherence				
MEDAS-14 score, points	7.44 ± 2.01	6.97 ± 2.06	7.81 ± 1.89	<0.001
Adherence category, n (%)				<0.001
Low MedDiet adherence MEDAS-14 < 9, n (%)	339 (68.3)	171 (77.7)	168 (60.9)	
High MedDiet adherence MEDAS-14 ≥ 9, n (%)	157 (31.7)	49 (22.3)	108 (39.1)	
Gambling-related variables				
CBJP, gambling problems, n (%)	18 (3.6)	14 (6.4)	4 (1.4)	0.004
Lie/Bet Questionnaire, suspected gambling problems, n (%)	12 (2.4)	10 (4.5)	2 (0.7)	0.006
Q1. Self-perceived gambling problems, n (%)	14 (2.8)	12 (5.5)	2 (0.7)	0.002
Q2. Feeling guilty about gambling, n (%)	21 (4.2)	18 (8.2)	3 (1.1)	<0.001
Q3. Tried to stop gambling unsuccessfully, n (%)	6 (1.2)	4 (1.8)	2 (0.7)	0.414
Q4. Took money from home to gamble/pay debts, n (%)	5 (1.0)	5 (2.3)	0 (0.0)	0.017
Q5. Lied to important people about gambling, n (%)	7 (1.4)	5 (2.3)	2 (0.7)	0.250
Q6. Need to bet increasing amounts of money, n (%)	15 (3.0)	13 (5.9)	2 (0.7)	<0.001

Data are presented as mean ± standard deviation or n (%), as appropriate. Differences between men and women were assessed using the independent-samples Student’s *t*-test or Welch’s *t*-test for continuous variables, depending on the homogeneity of variances, and Pearson’s chi-square test or Fisher’s exact test for categorical variables, as appropriate. For CBJP and Lie/Bet, values represent the number and percentage of participants with a positive screen; for Q1–Q6, values represent affirmative responses to the corresponding item. Abbreviations: MEDAS-14, 14-item Mediterranean Diet Adherence Screener; MedDiet, Mediterranean diet; CBJP, Cuestionario Breve de Juego Patológico; Q, question.

**Table 2:** Baseline characteristics of the study population according to Mediterranean diet adherence.

Variable	Low-to-Moderate MedDiet Adherence, MEDAS-14 < 9 (n = 339)	High MedDiet Adherence, MEDAS-14 ≥ 9 (n = 157)	*p*-Value
Sociodemographic characteristics			
Age, years	26.29 ± 4.53	27.28 ± 4.06	0.015
Marital status, n (%)			0.580
Married or partnered	62 (18.3)	32 (20.4)	
Not married or not partnered	277 (81.7)	125 (79.6)	
Educational level, n (%)			<0.001
University	185 (54.6)	116 (73.9)	
Non-university studies	154 (45.4)	41 (26.1)	
Lifestyle			
Alcohol intake, g/week	52.19 ± 74.03	41.88 ± 73.36	0.149
Sitting time, hours/week	55.17 ± 20.21	54.16 ± 18.86	0.600
Pittsburgh Sleep Quality Index score, points	5.83 ± 2.94	6.21 ± 3.23	0.200
Mediterranean diet adherence			
MEDAS-14 score, points	6.40 ± 1.46	9.69 ± 0.84	<0.001
Gambling-related variables			
CBJP, gambling problems, n (%)	15 (4.4)	3 (1.9)	0.164
Lie/Bet Questionnaire, suspected gambling problems, n (%)	10 (2.9)	2 (1.3)	0.355
Q1. Self-perceived gambling problems, n (%)	12 (3.5)	2 (1.3)	0.243
Q2. Feeling guilty about gambling, n (%)	19 (5.6)	2 (1.3)	0.026
Q3. Tried to stop gambling unsuccessfully, n (%)	4 (1.2)	2 (1.3)	1.000
Q4. Took money from home to gamble/pay debts, n (%)	4 (1.2)	1 (0.6)	1.000
Q5. Lied to important people about gambling, n (%)	6 (1.8)	1 (0.6)	0.441
Q6. Need to bet increasing amounts of money, n (%)	13 (3.8)	2 (1.3)	0.162

Data are presented as mean ± standard deviation or n (%), as appropriate. Participants were classified as having low-to-moderate MedDiet adherence (MEDAS-14 < 9) or high adherence (MEDAS-14 ≥ 9). Differences between groups were assessed using the independent-samples Student’s *t*-test or Welch’s *t*-test for continuous variables, according to the homogeneity of variances, and Pearson’s chi-square test or Fisher’s exact test for categorical variables, as appropriate. For CBJP and Lie/Bet, values represent the number and percentage of participants with a positive screen; for Q1–Q6, values represent affirmative responses to the corresponding item. Abbreviations: MEDAS-14, 14-item Mediterranean Diet Adherence Screener; MedDiet, Mediterranean diet; CBJP, Cuestionario Breve de Juego Patológico; Q, question.

## Data Availability

The data supporting the findings of this study are available on Zenodo at https://zenodo.org/records/16910594 under DOI 10.5281/zenodo.16910594 (accessed on 20 February 2026).

## Supplementary Materials

The following supporting information can be downloaded at https://www.mdpi.com/article/10.3390/nu18172919/s1: Table S1: Strengthening the Reporting of Observational Studies in Epidemiology (STROBE) checklist; Table S2: Collinearity diagnostics for the multiple linear regression models assessing associations between gambling-related indicators and MEDAS-14 score; Table S3: Sex-interaction analyses for associations between gambling-related indicators and MEDAS-14 score in Model 1 and the fully adjusted Model 2; Table S4: Exploratory sex-stratified associations between gambling-related indicators and MEDAS-14 score among women; Table S5: Pairwise overlap among positive gambling-related screening indicators (N = 496); Table S6: Model fit for linear regression analyses of gambling-related indicators and MEDAS-14 score.

## Abbreviations

APISAL, Salamanca Primary Care Research Unit; BCa, bias-corrected and accelerated; BMI, body mass index; CBJP, Cuestionario Breve de Juego Patológico (Short Pathological Gambling Questionnaire); CEIm, Research Ethics Committee for Medicinal Products; CERV, Video Game-Related Experiences Questionnaire; CI, confidence interval; CIUS, Compulsive Internet Use Scale; EDAS-18, Smartphone Addiction Scale; FDR, false discovery rate; IBSAL, Biomedical Research Institute of Salamanca; ISCIII, Carlos III Health Institute; MEDAS-13, modified 13-item Mediterranean Diet Adherence Screener; MEDAS-14, 14-item Mediterranean Diet Adherence Screener; MedDiet, Mediterranean diet; MSQ, Marshall Sitting Questionnaire; PREDIMED, PREvención con DIeta MEDiterránea (Prevention with Mediterranean Diet); PSQI, Pittsburgh Sleep Quality Index; Q1-Q6, Questions 1–6; RICAPPS, Research Network on Chronicity, Primary Care and Health Promotion; SACyL, Regional Health Management of Castilla y León; SD, standard deviation; SE, standard error; semFYC, Spanish Society of Family and Community Medicine; SPSS, Statistical Package for the Social Sciences; STROBE, Strengthening the Reporting of Observational Studies in Epidemiology; VIF, variance inflation factor.

## Appendix A

The members of the EVA-Adic Group are: Manuel A. Gómez Marcos, Luis García-Ortiz, Emiliano Rodríguez-Sánchez, Cristina Lugones-Sánchez, Olaya Tamayo-Morales, Susana González-Sánchez, Leticia Gómez-Sánchez, Sara M. Vicente-Gabriel, Alberto Vicente-Prieto, Sandra Conde-Martín, Marta Gómez-Sánchez, Elena Navarro Matias, Carmen Patino-Alonso, José A. Maderuelo-Fernández, Angela de Cabo-Laso, Benigna Sánchez-Salgado, and Cristina Saldaña-Ruiz.
