# Supplementary material for: Mediterranean Diet Adherence and Screening Indicators of Gambling-Related Problems in a Sample of Young Adults: An Exploratory Cross-Sectional Analysis of the EVA-Adic Study
Source: Nutrients. 2026 Sep 6;18(17):2919. doi: 10.3390/nu18172919 (PMC13567718; doi:10.3390/nu18172919)
Supplement: Supplementary file 1 [file nutrients-18-02919-s001.zip › nutrients-4528533-supplementary.pdf]

Supplementary material

Supplementary Table S1. STROBE Checklist for Cross-Sectional Studies. Manuscript: Mediterranean Diet Adherence and Screening Indicators of Gambling-Related Problems in a Sample of Young Adults: An Exploratory Cross-Sectional Analysis of the EVA-Adic Study. Target journal: Nutrients (MDPI) | Study design: Cross-sectional observational study | Analytical sample: n = 496

Section/topic	Item No.	STROBE recommendation	Reported on page/section
Title and abstract	1a	Indicate the study's design with a commonly used term in the title or the abstract.	p. 1, Abstract (Methods: "A cross-sectional analysis…").
Title and abstract	1b	Provide in the abstract an informative and balanced summary of what was done and what was found.	pp. 1–2, Abstract.
Introduction: Background/rationale	2	Explain the scientific background and rationale for the investigation being reported.	pp. 2–4, Section 1. Introduction.
Introduction: Objectives	3	State specific objectives, including any prespecified hypotheses.	p. 4, final paragraph of the Introduction.
Methods: Study design	4	Present key elements of the study design early in the paper.	p. 4, Section 2.1. Study Design.
Methods: Setting	5	Describe the setting, locations, and relevant dates	pp. 4–6, Sections 2.1–2.2 and beginning of Section 2.4.
Methods: Participants	6	Give the eligibility criteria, and the sources and methods of selection of participants.	pp. 4–6, Section 2.2 and Figure 1.
Methods: Variables	7	Clearly define all outcomes, exposures, predictors, potential confounders, and effect modifiers. Give diagnostic criteria, if applicable.	pp. 6–9, Sections 2.4.1–2.5.
Methods: Data sources/measurement	8	For each variable of interest, give sources of data and details of methods of assessment (measurement). Describe comparability of assessment methods if there is more than one group.	pp. 6–9, Sections 2.4–2.5.
Methods: Bias	9	Describe any efforts to address potential sources of bias.	Sections 2.1–2.2 and 2.5 (recruitment procedures, standardized assessments, separate regression models, robustness analyses, and quality control); Strengths and Limitations subsection.
Methods: Study size	10	Explain how the study size was arrived at.	p. 6, Section 2.3. Study Size.
Methods: Quantitative variables	11	Explain how quantitative variables were handled in the analyses. If applicable, describe which groupings were chosen and why.	pp. 7–9, Sections 2.4.3 and 2.5.
Methods: Statistical methods	12a	Describe all statistical methods, including those used to control for confounding.	pp. 7–9, Section 2.5. Statistical Analysis.
Methods: Statistical methods	12b	Describe any methods used to examine subgroups and interactions.	pp. 8–9, Section 2.5.
Methods: Statistical methods	12c	Explain how missing data were addressed.	pp. 4 and 7–8, Sections 2.2 and 2.5; Figure 1.
Methods: Statistical methods	12d	If applicable, describe analytical methods taking account of the sampling strategy.	Not applicable: consecutive sampling was used without a complex survey design, weighting, or clustering.
Methods: Statistical methods	12e	Describe any sensitivity analyses.	Section 2.5 (Statistical Analysis) and Results (sensitivity and robustness analyses).
Results: Participants	13a	Report the numbers of individuals at each stage of the study—for example, numbers potentially eligible, examined for eligibility, confirmed eligible, included in the study, completing follow-up, and analysed.	pp. 4–6, Section 2.2 and Figure 1.
Results: Participants	13b	Give reasons for non-participation at each stage.	Section 2.2 (open recruitment; a conventional denominator for non-participation was not available).
Results: Participants	13c	Consider use of a flow diagram.	p. 5, Figure 1.

Supplementary material

Section/topic	Item No.	STROBE recommendation	Reported on page/section
Results: Descriptive data	14a	Give characteristics of study participants (for example, demographic, clinical, and social) and information on exposures and potential confounders.	pp. 10–13, Tables 1 and 2.
Results: Descriptive data	14b	Indicate the number of participants with missing data for each variable of interest.	pp. 4 and 7–8, Sections 2.2 and 2.5; Figure 1.
Results: Outcome data	15	Report numbers of outcome events or summary measures.	pp. 10–14, Tables 1–2 and Figures 2–3.
Results: Main results	16a	Give unadjusted estimates and, if applicable, confounder-adjusted estimates and their precision (for example, 95% confidence intervals). Make clear which confounders were adjusted for and why they were included.	pp. 8–9, statistical methods; pp. 13–16, Figures 2–5 and figure legends.
Results: Main results	16b	Report category boundaries when continuous variables were categorised.	pp. 7–9 and pp. 10–13.
Results: Main results	16c	If relevant, consider translating estimates of relative risk into absolute risk for a meaningful time period.	Not applicable: this is a cross-sectional study and the primary outcome is a continuous score.
Results: Other analyses	17	Report other analyses performed—for example, analyses of subgroups and interactions, and sensitivity analyses.	Results (subgroup, interaction, multiplicity, and sensitivity analyses); Supplementary Tables S3–S5.
Discussion: Key results	18	Summarise key results with reference to study objectives.	p. 17, first paragraph of Section 4. Discussion.
Discussion: Limitations	19	Discuss limitations of the study, taking into account sources of potential bias or imprecision. Discuss both the direction and magnitude of any potential bias.	pp. 19–20, strengths and limitations subsection.
Discussion: Interpretation	20	Give a cautious overall interpretation of results considering objectives, limitations, multiplicity of analyses, results from similar studies, and other relevant evidence.	pp. 17–20, Section 4. Discussion.
Discussion: Generalisability	21	Discuss the generalisability (external validity) of the study results.	p. 20, final part of the limitations subsection.
Other information: Funding	22	Give the source of funding and the role of the funders for the present study and, if applicable, for the original study on which the present article is based.	p. 21, Funding statement.

N/A, not applicable.

Supplementary material

Supplementary Table S2. Collinearity diagnostics for multiple linear regression models assessing associations between gambling-related indicators and MEDAS-14 score

Panel A. Summary of collinearity diagnostics

Gambling-related indicator	Model 1 Minimum tolerance	Model 1 Maximum VIF	Model 2 Minimum tolerance	Model 2 Maximum VIF	Model 1 Gambling predictor tolerance	Model 1 Gambling predictor VIF	Model 2 Gambling predictor tolerance	Model 2 Gambling predictor VIF	Assessment
Gambling problems	0.929	1.076	0.663	1.509	0.962	1.039	0.957	1.045	No relevant multicollinearity
Suspected gambling problems	0.932	1.073	0.665	1.504	0.951	1.051	0.939	1.065	No relevant multicollinearity
Q1. Self-perceived gambling problems	0.932	1.073	0.666	1.502	0.959	1.043	0.953	1.049	No relevant multicollinearity
Q2. Feeling guilty about gambling	0.923	1.084	0.657	1.523	0.944	1.059	0.929	1.077	No relevant multicollinearity
Q3. Tried to stop gambling, unsuccessfully	0.932	1.073	0.664	1.506	0.972	1.029	0.961	1.041	No relevant multicollinearity
Q4. Took household money to gamble/pay debts	0.932	1.073	0.667	1.500	0.971	1.030	0.969	1.032	No relevant multicollinearity
Q5. Lied to important people about gambling	0.932	1.074	0.664	1.505	0.963	1.038	0.956	1.046	No relevant multicollinearity
Q6. Need to bet increasing amounts of money	0.928	1.078	0.663	1.508	0.966	1.036	0.964	1.037	No relevant multicollinearity

Panel B. Detailed tolerance and VIF values

Model	Gambling-related indicator examined	Predictor included in regression	Tolerance	VIF
Model 1	Gambling problems	Age	0.929	1.076
Model 1	Gambling problems	Sex	0.938	1.066
Model 1	Gambling problems	Alcohol intake (g/week)	0.963	1.038
Model 1	Gambling problems	Pittsburgh Sleep Quality Index score	0.972	1.029
Model 1	Gambling problems	Sitting time (h/week)	0.948	1.055
Model 1	Gambling problems	Gambling problems	0.962	1.039
Model 1	Suspected gambling problems	Age	0.932	1.073
Model 1	Suspected gambling problems	Sex	0.936	1.068
Model 1	Suspected gambling problems	Alcohol intake (g/week)	0.971	1.030

Supplementary material

Model	Gambling-related indicator examined	Predictor included in regression	Tolerance	VIF
Model 1	Suspected gambling problems	Pittsburgh Sleep Quality Index score	0.960	1.041
Model 1	Suspected gambling problems	Sitting time (h/week)	0.937	1.067
Model 1	Suspected gambling problems	Suspected gambling problems	0.951	1.051
Model 1	Q1. Self-perceived gambling problems	Age	0.932	1.073
Model 1	Q1. Self-perceived gambling problems	Sex	0.933	1.071
Model 1	Q1. Self-perceived gambling problems	Alcohol intake (g/week)	0.966	1.035
Model 1	Q1. Self-perceived gambling problems	Pittsburgh Sleep Quality Index score	0.968	1.034
Model 1	Q1. Self-perceived gambling problems	Sitting time (h/week)	0.945	1.058
Model 1	Q1. Self-perceived gambling problems	Q1. Self-perceived gambling problems	0.959	1.043
Model 1	Q2. Feeling guilty about gambling	Age	0.923	1.084
Model 1	Q2. Feeling guilty about gambling	Sex	0.924	1.082
Model 1	Q2. Feeling guilty about gambling	Alcohol intake (g/week)	0.974	1.027
Model 1	Q2. Feeling guilty about gambling	Pittsburgh Sleep Quality Index score	0.967	1.034
Model 1	Q2. Feeling guilty about gambling	Sitting time (h/week)	0.947	1.056
Model 1	Q2. Feeling guilty about gambling	Q2. Feeling guilty about gambling	0.944	1.059
Model 1	Q3. Tried to stop gambling, unsuccessfully	Age	0.932	1.073
Model 1	Q3. Tried to stop gambling, unsuccessfully	Sex	0.949	1.053
Model 1	Q3. Tried to stop gambling, unsuccessfully	Alcohol intake (g/week)	0.962	1.039
Model 1	Q3. Tried to stop gambling, unsuccessfully	Pittsburgh Sleep Quality Index score	0.967	1.034
Model 1	Q3. Tried to stop gambling, unsuccessfully	Sitting time (h/week)	0.946	1.057
Model 1	Q3. Tried to stop gambling, unsuccessfully	Q3. Tried to stop gambling, unsuccessfully	0.972	1.029
Model 1	Q4. Took household money to gamble/pay debts	Age	0.932	1.073
Model 1	Q4. Took household money to gamble/pay debts	Sex	0.938	1.066
Model 1	Q4. Took household money to gamble/pay debts	Alcohol intake (g/week)	0.975	1.026
Model 1	Q4. Took household money to gamble/pay debts	Pittsburgh Sleep Quality Index score	0.968	1.033
Model 1	Q4. Took household money to gamble/pay debts	Sitting time (h/week)	0.942	1.061
Model 1	Q4. Took household money to gamble/pay debts	Q4. Took household money to gamble/pay debts	0.971	1.030
Model 1	Q5. Lied to important people about gambling	Age	0.932	1.074
Model 1	Q5. Lied to important people about gambling	Sex	0.949	1.054
Model 1	Q5. Lied to important people about gambling	Alcohol intake (g/week)	0.949	1.054
Model 1	Q5. Lied to important people about gambling	Pittsburgh Sleep Quality Index score	0.973	1.027

Supplementary material

Model	Gambling-related indicator examined	Predictor included in regression	Tolerance	VIF
Model 1	Q5. Lied to important people about gambling	Sitting time (h/week)	0.947	1.056
Model 1	Q5. Lied to important people about gambling	Q5. Lied to important people about gambling	0.963	1.038
Model 1	Q6. Need to bet increasing amounts of money	Age	0.928	1.078
Model 1	Q6. Need to bet increasing amounts of money	Sex	0.932	1.073
Model 1	Q6. Need to bet increasing amounts of money	Alcohol intake (g/week)	0.973	1.028
Model 1	Q6. Need to bet increasing amounts of money	Pittsburgh Sleep Quality Index score	0.974	1.027
Model 1	Q6. Need to bet increasing amounts of money	Sitting time (h/week)	0.948	1.055
Model 1	Q6. Need to bet increasing amounts of money	Q6. Need to bet increasing amounts of money	0.966	1.036
Model 2 (fully adjusted)	Gambling problems	Age	0.663	1.509
Model 2 (fully adjusted)	Gambling problems	Sex	0.909	1.100
Model 2 (fully adjusted)	Gambling problems	Alcohol intake (g/week)	0.962	1.039
Model 2 (fully adjusted)	Gambling problems	Pittsburgh Sleep Quality Index score	0.959	1.042
Model 2 (fully adjusted)	Gambling problems	Sitting time (h/week)	0.943	1.061
Model 2 (fully adjusted)	Gambling problems	Marital status	0.835	1.198
Model 2 (fully adjusted)	Gambling problems	Educational level	0.810	1.235
Model 2 (fully adjusted)	Gambling problems	Gambling problems	0.957	1.045
Model 2 (fully adjusted)	Suspected gambling problems	Age	0.665	1.504
Model 2 (fully adjusted)	Suspected gambling problems	Sex	0.909	1.101
Model 2 (fully adjusted)	Suspected gambling problems	Alcohol intake (g/week)	0.970	1.031
Model 2 (fully adjusted)	Suspected gambling problems	Pittsburgh Sleep Quality Index score	0.950	1.052
Model 2 (fully adjusted)	Suspected gambling problems	Sitting time (h/week)	0.933	1.072

Supplementary material

Model	Gambling-related indicator examined	Predictor included in regression	Tolerance	VIF
Model 2 (fully adjusted)	Suspected gambling problems	Marital status	0.835	1.197
Model 2 (fully adjusted)	Suspected gambling problems	Educational level	0.804	1.244
Model 2 (fully adjusted)	Suspected gambling problems	Suspected gambling problems	0.939	1.065
Model 2 (fully adjusted)	Q1. Self-perceived gambling problems	Age	0.666	1.502
Model 2 (fully adjusted)	Q1. Self-perceived gambling problems	Sex	0.905	1.105
Model 2 (fully adjusted)	Q1. Self-perceived gambling problems	Alcohol intake (g/week)	0.965	1.036
Model 2 (fully adjusted)	Q1. Self-perceived gambling problems	Pittsburgh Sleep Quality Index score	0.956	1.046
Model 2 (fully adjusted)	Q1. Self-perceived gambling problems	Sitting time (h/week)	0.940	1.063
Model 2 (fully adjusted)	Q1. Self-perceived gambling problems	Marital status	0.835	1.197
Model 2 (fully adjusted)	Q1. Self-perceived gambling problems	Educational level	0.810	1.235
Model 2 (fully adjusted)	Q1. Self-perceived gambling problems	Q1. Self-perceived gambling problems	0.953	1.049
Model 2 (fully adjusted)	Q2. Feeling guilty about gambling	Age	0.657	1.523
Model 2 (fully adjusted)	Q2. Feeling guilty about gambling	Sex	0.898	1.113
Model 2 (fully adjusted)	Q2. Feeling guilty about gambling	Alcohol intake (g/week)	0.974	1.027
Model 2 (fully adjusted)	Q2. Feeling guilty about gambling	Pittsburgh Sleep Quality Index score	0.956	1.046
Model 2 (fully adjusted)	Q2. Feeling guilty about gambling	Sitting time (h/week)	0.942	1.061
Model 2 (fully adjusted)	Q2. Feeling guilty about gambling	Marital status	0.835	1.198
Model 2 (fully adjusted)	Q2. Feeling guilty about gambling	Educational level	0.802	1.247

Supplementary material

Model	Gambling-related indicator examined	Predictor included in regression	Tolerance	VIF
Model 2 (fully adjusted)	Q2. Feeling guilty about gambling	Q2. Feeling guilty about gambling	0.929	1.077
Model 2 (fully adjusted)	Q3. Tried to stop gambling, unsuccessfully	Age	0.664	1.506
Model 2 (fully adjusted)	Q3. Tried to stop gambling, unsuccessfully	Sex	0.918	1.089
Model 2 (fully adjusted)	Q3. Tried to stop gambling, unsuccessfully	Alcohol intake (g/week)	0.961	1.041
Model 2 (fully adjusted)	Q3. Tried to stop gambling, unsuccessfully	Pittsburgh Sleep Quality Index score	0.956	1.046
Model 2 (fully adjusted)	Q3. Tried to stop gambling, unsuccessfully	Sitting time (h/week)	0.941	1.062
Model 2 (fully adjusted)	Q3. Tried to stop gambling, unsuccessfully	Marital status	0.834	1.198
Model 2 (fully adjusted)	Q3. Tried to stop gambling, unsuccessfully	Educational level	0.805	1.243
Model 2 (fully adjusted)	Q3. Tried to stop gambling, unsuccessfully	Q3. Tried to stop gambling, unsuccessfully	0.961	1.041
Model 2 (fully adjusted)	Q4. Took household money to gamble/pay debts	Age	0.667	1.500
Model 2 (fully adjusted)	Q4. Took household money to gamble/pay debts	Sex	0.907	1.103
Model 2 (fully adjusted)	Q4. Took household money to gamble/pay debts	Alcohol intake (g/week)	0.975	1.026
Model 2 (fully adjusted)	Q4. Took household money to gamble/pay debts	Pittsburgh Sleep Quality Index score	0.955	1.047
Model 2 (fully adjusted)	Q4. Took household money to gamble/pay debts	Sitting time (h/week)	0.937	1.067
Model 2 (fully adjusted)	Q4. Took household money to gamble/pay debts	Marital status	0.834	1.199
Model 2 (fully adjusted)	Q4. Took household money to gamble/pay debts	Educational level	0.814	1.229
Model 2 (fully adjusted)	Q4. Took household money to gamble/pay debts	Q4. Took household money to gamble/pay debts	0.969	1.032
Model 2 (fully adjusted)	Q5. Lied to important people about gambling	Age	0.664	1.505

Supplementary material

Model	Gambling-related indicator examined	Predictor included in regression	Tolerance	VIF
Model 2 (fully adjusted)	Q5. Lied to important people about gambling	Sex	0.917	1.090
Model 2 (fully adjusted)	Q5. Lied to important people about gambling	Alcohol intake (g/week)	0.948	1.055
Model 2 (fully adjusted)	Q5. Lied to important people about gambling	Pittsburgh Sleep Quality Index score	0.961	1.041
Model 2 (fully adjusted)	Q5. Lied to important people about gambling	Sitting time (h/week)	0.941	1.062
Model 2 (fully adjusted)	Q5. Lied to important people about gambling	Marital status	0.831	1.203
Model 2 (fully adjusted)	Q5. Lied to important people about gambling	Educational level	0.812	1.231
Model 2 (fully adjusted)	Q5. Lied to important people about gambling	Q5. Lied to important people about gambling	0.956	1.046
Model 2 (fully adjusted)	Q6. Need to bet increasing amounts of money	Age	0.663	1.508
Model 2 (fully adjusted)	Q6. Need to bet increasing amounts of money	Sex	0.902	1.108
Model 2 (fully adjusted)	Q6. Need to bet increasing amounts of money	Alcohol intake (g/week)	0.972	1.028
Model 2 (fully adjusted)	Q6. Need to bet increasing amounts of money	Pittsburgh Sleep Quality Index score	0.961	1.041
Model 2 (fully adjusted)	Q6. Need to bet increasing amounts of money	Sitting time (h/week)	0.943	1.061
Model 2 (fully adjusted)	Q6. Need to bet increasing amounts of money	Marital status	0.835	1.197
Model 2 (fully adjusted)	Q6. Need to bet increasing amounts of money	Educational level	0.813	1.230
Model 2 (fully adjusted)	Q6. Need to bet increasing amounts of money	Q6. Need to bet increasing amounts of money	0.964	1.037

Tolerance and variance inflation factor (VIF) values were used to assess multicollinearity; VIF values <2 were considered to indicate low collinearity. Model 1 included age, sex, alcohol intake (g/week), Pittsburgh Sleep Quality Index score, sitting time, and one gambling-related indicator entered separately. Model 2 additionally included marital status and educational level. Across all Model 1 regressions, tolerance ranged from 0.923 to 0.975 and VIF from 1.026 to 1.084. Across all Model 2 regressions, tolerance ranged from 0.657 to 0.975 and VIF from 1.026 to 1.523. No relevant multicollinearity was identified.

Supplementary material

Supplementary Table S3. Sex-interaction analyses for associations between gambling-related indicators and MEDAS-14 score in Model 1 and the fully adjusted Model 2

Gambling-related indicator	Model 1				Model 2 (fully adjusted)			
	B (SE)	95% CI	p for interaction	FDR q	B (SE)	95% CI	p for interaction	FDR q
Gambling problems	2.120 (1.127)	-0.095 to 4.335	0.061	0.172	2.284 (1.121)	0.080 to 4.487	**0.042**	0.101
Suspected gambling problems	2.459 (1.552)	-0.592 to 5.509	0.114	0.172	2.651 (1.543)	-0.380 to 5.682	0.086	0.101
Q1. Self-perceived gambling problems	2.330 (1.525)	-0.666 to 5.325	0.127	0.172	2.617 (1.516)	-0.362 to 5.595	0.085	0.101
Q2. Feeling guilty about gambling	2.227 (1.245)	-0.219 to 4.673	0.074	0.172	2.518 (1.244)	0.073 to 4.963	**0.044**	0.101
Q3. Tried to stop gambling, unsuccessfully	2.823 (1.727)	-0.571 to 6.217	0.103	0.172	2.999 (1.714)	-0.368 to 6.367	0.081	0.101
Q4. Took household money to gamble/pay debts	NE	NE	NE	NE	NE	NE	NE	NE
Q5. Lied to important people about gambling	2.409 (1.661)	-0.853 to 5.672	0.147	0.172	2.846 (1.650)	-0.395 to 6.087	0.085	0.101
Q6. Need to bet increasing amounts of money	2.009 (1.500)	-0.938 to 4.957	0.181	0.181	2.038 (1.489)	-0.888 to 4.964	0.172	0.172

Values are unstandardized regression coefficients (B) for the sex x gambling-related indicator interaction term, with standard errors (SE), 95% confidence intervals (CI), nominal p values, and Benjamini-Hochberg false discovery rate (FDR)-adjusted q values. Model 1 included age, sex, alcohol intake (g/week), Pittsburgh Sleep Quality Index score, sitting time, the corresponding gambling-related indicator, and the interaction term. Model 2 additionally included marital status and educational level. Sex was coded as 0 = men and 1 = women, and gambling-related indicators as 0 = absence and 1 = presence; therefore, men without the corresponding indicator were the reference category. FDR correction was applied separately to the seven estimable interaction tests within each model. No Model 1 interaction was nominally significant. In Model 2, nominal interactions were observed for gambling problems and Q2, but neither remained statistically significant after FDR correction. The Q4 interaction was not estimable because no women endorsed this indicator. N = 496. NE, not estimable.

Supplementary material

Supplementary Table S4. Exploratory sex-stratified associations between gambling-related indicators and MEDAS-14 score among women

Gambling-related indicator	Positive women, n	Model 1			Model 2 (fully adjusted)		
		B	95% CI	p value	B	95% CI	p value
Gambling problems	4	0.736	-1.188 to 2.660	0.452	1.025	-0.888 to 2.937	0.293
Suspected gambling problems	2	0.685	-2.101 to 3.470	0.629	1.062	-1.699 to 3.822	0.450
Q1. Self-perceived gambling problems	2	0.685	-2.101 to 3.470	0.629	1.062	-1.699 to 3.822	0.450
Q2. Feeling guilty about gambling	3	0.531	-1.725 to 2.788	0.643	1.084	-1.176 to 3.345	0.346
Q3. Tried to stop gambling, unsuccessfully	2	0.685	-2.101 to 3.470	0.629	1.062	-1.699 to 3.822	0.450
Q4. Took household money to gamble/pay debts	0	NE	—	—	NE	—	—
Q5. Lied to important people about gambling	2	0.685	-2.101 to 3.470	0.629	1.062	-1.699 to 3.822	0.450
Q6. Need to bet increasing amounts of money	2	0.762	-1.865 to 3.389	0.569	0.949	-1.653 to 3.551	0.473

Values are unstandardized regression coefficients (B) with 95% confidence intervals (CI). Each gambling-related indicator was examined in a separate model. Model 1 was adjusted for age, alcohol intake (g/week), Pittsburgh Sleep Quality Index score, and sitting time. Model 2 additionally included marital status and educational level. Q4 could not be estimated because no women endorsed this indicator. Given the very small number of women endorsing several indicators, these sex-stratified analyses are exploratory and should not be interpreted as confirmatory evidence of sex-specific associations. MEDAS-14, 14-item Mediterranean Diet Adherence Screener; CI, confidence interval; NE, not estimable.

Supplementary material

Supplementary Table S5. Pairwise overlap among positive gambling-related screening indicators (N = 496)

Indicator	Positive n	Gambling problems	Suspected gambling problems	Q1	Q2	Q3	Q4	Q5	Q6
**Gambling problems**	18	—	8	10	12	4	2	7	15
**Suspected gambling problems**	12	8	—	11	11	6	4	5	6
Q1. Self-perceived gambling problems	14	10	11	—	10	5	4	7	7
Q2. Feeling guilty about gambling	21	12	11	10	—	5	3	5	10
Q3. Tried to stop gambling unsuccessfully	6	4	6	5	5	—	2	3	2
Q4. Took household money to gamble/pay debts	5	2	4	4	3	2	—	1	2
Q5. Lied to important people about gambling	7	7	5	7	5	3	1	—	4
Q6. Need to bet increasing amounts of money	15	15	6	7	10	2	2	4	—

Note. Values represent the number of participants who were simultaneously positive for the two corresponding gambling-related indicators. "Positive n" indicates the total number of participants positive for each indicator. Because the composite screening measures and individual items represent related and overlapping constructs, these indicators should not be interpreted as statistically independent sources of evidence.

Abbreviations: Q, question.

Supplementary material

Supplementary Table S6. Model fit for linear regression analyses of gambling-related indicators and MEDAS-14 score

Gambling-related indicator	Minimally adjusted model R^2	Model 1 R^2	Model 2 (fully adjusted) R^2
Gambling problems	0.060	0.064	0.083
Suspected gambling problems	0.059	0.065	0.083
Q1. Self-perceived gambling problems	0.061	0.066	0.085
Q2. Feeling guilty about gambling	0.067	0.073	0.090
Q3. Tried to stop gambling, unsuccessfully	0.056	0.060	0.080
Q4. Took money from home to gamble/pay debts	0.054	0.059	0.079
Q5. Lied to important people about gambling	0.056	0.060	0.081
Q6. Need to bet more and more money	0.061	0.065	0.085

R^2 values refer to the proportion of variance in MEDAS-14 score explained by the complete regression model and should not be interpreted as the variance uniquely attributable to the gambling-related indicator. The minimally adjusted model included age, sex, and the corresponding gambling-related indicator. Model 1 additionally included alcohol consumption (g/week), Pittsburgh Sleep Quality Index score, and sitting time. Model 2 additionally included marital status and educational level. Each gambling-related indicator was examined in a separate regression model. N = 496. MEDAS-14, 14-item Mediterranean Diet Adherence Screener.
